# Behavioral parent training for disruptive behaviors in school-age children with autism: Secondary outcomes of a randomized controlled trial

**DOI:** 10.1007/s00787-026-02985-w

**Published:** 2026-02-20

**Authors:** Tycho J. Dekkers, Mandy Woelk, Simone Breider, Pieter J. Hoekstra, Barbara J. van den Hoofdakker, Annelies de Bildt

**Affiliations:** 1 Accare Child Study Center, Groningen, The Netherlands; 2 Department of Psychiatry, University Medical Center Groningen, University of Groningen, Groningen, The Netherlands; 3 Levvel, Academic Center for Specialized Youthcare, Amsterdam, The Netherlands; 4 The Research School of Behavioural and Cognitive Neurosciences, University of Groningen, Groningen, The Netherlands; 5 Department of Clinical Psychology and Experimental Psychopathology, University of Groningen, Groningen, The Netherlands

**Keywords:** Behavioral parent training, Autism, Disruptive behavior, Parenting

## Abstract

Children with autism often show disruptive behavior problems, which may cause significant impairment. Behavioral parent training is an effective intervention for other children with disruptive behavior, but research in children with autism is relatively scarce. We here report the secondary outcomes of a three-arm randomized controlled trial, comparing face-to-face and blended parent training to a waitlist control condition for children with autism and disruptive behavior. We previously found that face-to-face, but not blended parent training, relative to waitlist control, significantly improved children’s noncompliance and irritability. Knowledge about its effects on parental functioning and other domains of children’s functioning is limited. Using linear regression analyses on an intention-to-treat basis, we investigated parent training effects on a range of parenting (parental satisfaction, parental efficacy, parenting stress, and lax, overreactive and verbose parenting styles) and child outcomes (hyperactivity, emotional problems, conduct problems, peer problems, prosocial behavior, and adaptive behavior). We found that face-to-face, but not blended parent training, improved parental self-efficacy and decreased overreactive parenting relative to the waitlist condition. We found no intervention effects of either parent training format on any of the other outcomes. Overall, our findings add to the evidence for face-to-face behavioral parent training as an effective intervention for disruptive behavior in children with autism by illustrating that not only children but also parents improve. This randomized controlled trial was registered in the Dutch Trial Register (#22,042).

## Introduction

Children with autism^1^ often show disruptive behavior problems, such as aggression, noncompliance, and irritability [21], which is associated with poorer family functioning [36]. For other children with disruptive behaviors, in particular children with attention deficit hyperactivity disorder (ADHD) or oppositional defiant disorder (ODD), behavioral parent training is the non-pharmacological treatment of first choice, given its strong and robust effects [10, 27]. For children with autism, two partially overlapping meta-analyses illustrate the potential of this intervention for the reduction of disruptive or hyperactive behaviors [30, 41].

With regard to behavioral parent training for disruptive behaviors in children with autism, effects on the parents themselves are relatively less explored. This is remarkable, as changes in parental behaviors and cognitions are supposed to mediate changes in behaviors of the child. A meta-analysis on behavioral parent training targeting disruptive or hyperactive behaviors in children with autism found small-to-medium positive effects on parenting stress and parenting sense of competence, but the included number of studies was low (seven and five, respectively; [41]). A more recent trial on Parent–Child Interaction Therapy (PCIT) for disruptive behavior in children with autism also found effects on parental stress [2], but another PCIT trial, as well as a trial on a digital behavioral parent training intervention for children with autism did not find such an effect [24, 49]. One study provided evidence for the supposed mediation model: reductions in child disruptive behavior were mediated by changes in negative, but not positive, parenting practices [13]. In sum, for children with autism , parenting seems to improve as a result of parent training, but the available literature is scarce, and most studies are limited by small samples.

Additionally, effects of behavioral parent training on other domains of functioning in children with autism are unclear. This is surprising, as children with autism often have co-occurring behavioral and emotional problems [17, 18] and lower levels of adaptive behaviors [22, 28], which can be challenging for parents. A few trials tested effects of parent training on such behaviors, and findings were mixed. Improvements in the social domain were found in some trials (e.g., social awareness [13]; social interaction [37]; adaptability [35, 38]), but not in others [3, 16]. Similarly, one trial reported decreases in children’s stereotypical behaviors [3], while another observed no change [16]. For hyperactivity, a small but significant effect was reported in a meta-analysis, although this finding was based on the synthesis of only three trials [41]. Finally, a recent meta-analysis on parent-implemented interventions for children with autism found positive effects on child outcomes like social skills, language and communication, and adaptive behavior, but most of these studies focused on preschoolers, and interventions were heterogeneous and mostly not focused on parenting [47]. In sum, the generalizability of the effects of parent training on the behavior of children with autism beyond the targeted disruptive behaviors remains uncertain.

In a recent randomized controlled trial, we compared the effects of face-to-face behavioral parent training as well as a therapist-assisted online (i.e., blended) version of the same program with a waitlist control condition on children’s noncompliance and irritability. We found that children in the face-to-face condition showed less noncompliance and irritability relative to children in the waitlist control group, whereas this was not the case for children receiving the blended program [8]. Findings were similar directly after the intervention and at a six-month follow-up assessment.

In the current study we aimed to determine to what extent the primary results of this trial extend to other outcomes, focusing specifically on two outcome domains. First, we tested whether individual behavioral parent training also led to improvements on a range of parenting outcomes, such as parents’ perceived parenting competence, stress, and general parenting style. Second, we investigated whether individual behavioral parent training affected more general parent-reported improvements in the behavior of the child beyond the decrease in noncompliance and irritability as described earlier. These included measures of psychosocial functioning such as hyperactivity, emotional problems, conduct problems, peer problems, and prosocial behaviors, as well as adaptive child behaviors in the domains of communication, daily living skills and socialization. Based upon the outcomes on our primary measures [8], we hypothesized that the face-to-face parent training program would be superior to waitlist on all outcome measures, while we did not anticipate effects of the blended training program.

## Methods

### Design

This two-center randomized controlled trial included parents of 97 children, randomized over three conditions: face-to-face parent training (*n* = 33), blended parent training (*n* = 33), or a waitlist control condition (*n* = 31). Details of the study design, procedure, and training content, have been published elsewhere [8] and are therefore only summarized in the current manuscript. For details on disruptive behavior outcomes, also see [8]. The trial has been registered in the Dutch Trial Register (see https://onderzoekmetmensen.nl/en/trial/22042.)

### Participants

**Fig. 1:**
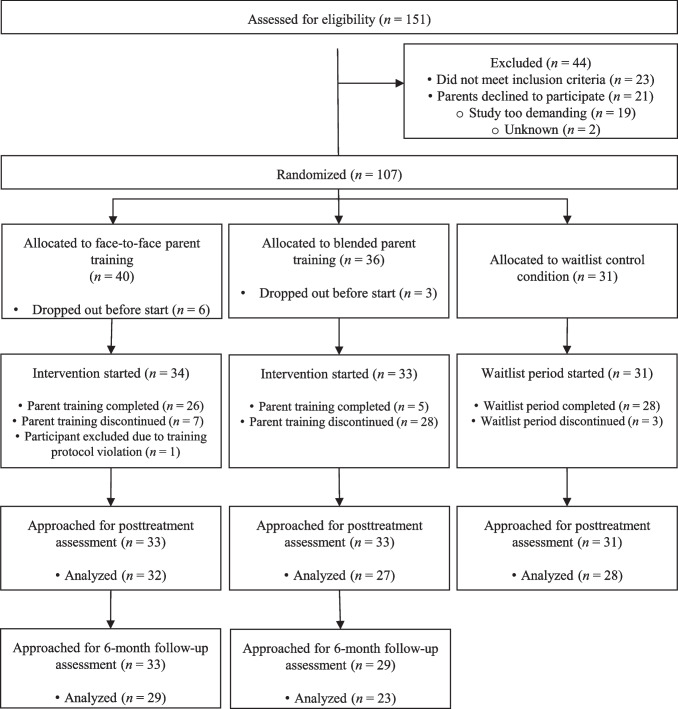
Participant Flow Chart

Recruitment took place between December 2014 and March 2018 from two outpatient centers for child and adolescent psychiatry in the Netherlands. Participants were included if the child: 1) had an Autism Spectrum Disorder diagnosis according to the DSM-IV-TR [4] or DSM-5[5]; 2) was aged between 4 and 13 years; 3) had a total IQ higher than 50; 4) did not use psychotropic medication or was on a stable dose for at least 6 weeks before study entry, with no expected changes during the study; and 5) had at least three parent-reported disruptive behaviors at home, as assessed by the list of target behaviors [44]. Furthermore, 6) at least one parent had to be willing to participate in the parent training; and 7) parents had to have a laptop or PC at their disposal. Exclusion criteria were the following: 1) the family required immediate intervention (e.g., families in which one of the parents was experiencing acute mental problems or in which the safety of the child could not be guaranteed); 2) parents had received parent training in the year prior to the study; and 3) the family intended to move to a region far from one of the study locations within six months. No restrictions were set to the child’s co-occurring mental health problems. For full details on participant flow, see Fig. 1. The study was approved by the Medical Ethics Review Committee of the University Medical Center Groningen.

### Procedure

Parents of potentially eligible children were invited by their clinician to participate in the study. If parents were interested to participate, we provided parents and children with more details about the study. If they agreed to participate, parents and children aged 12 or older signed informed consent.

Parents completed the baseline questionnaire online (also see Measures below). Participants were randomized after pre-assessment, and in the two training conditions families started with the parent training on average 47 days (*SD* = 20.38 days) after completion of the baseline questionnaire. At the start of the training, parents selected three to five problem behaviors and situations to be targeted in the parent training. Both the face-to-face and the blended training consisted of four phases: 1) introduction and psychoeducation; 2) techniques to change antecedents of problem behavior; 3) techniques to change consequences of problem behavior; and 4) evaluation and generalization.

Participants in the face-to-face training condition received the full training in a live setting with the same therapist, with sessions lasting between 45 and 60 min. Participants in the blended training condition completed the training through an online program with different modules in a set order. They received written feedback from a therapist on their exercises. In addition, they attended at least two face-to-face sessions (45–60 min) with a therapist, to evaluate their progress. Participants in the waitlist control condition went through a waitlist period of 20 weeks. In all conditions, children and parents were allowed to receive other (mental) health care, except for behavioral parenting interventions directed at the child’s behavior.

### Measures

All measures were completed by the parents. For all but one participant, the parent who spent most time with the child attended the parent training (either alone or together with another parent) and the assessments of these parents were used for the analyses. For the outcome measures, we only used the subscales of each measure.

#### Demographics

o **Child.** Parents reported their child’s age, sex, birth country, school type, IQ, autism severity, co-occurring mental health problems, and mental health care use (directed at the child).
o **Parent.** Information was collected regarding the parent’s age, relation to the child, working situation, family composition, mental health care use (directed at the child), and several (self-reported) behaviors that will be published elsewhere.

#### Parent-related Measures

##### Parenting sense of competence

The Parenting Sense of Competence scale (PSOC [12]) contains two subscales: Parenting Satisfaction and Parenting Efficacy. The Parenting Satisfaction subscale consists of nine items and assesses the extent to which parents feel satisfied in their role as a parent (e.g., “Sometimes I feel like I’m not getting anything done as a parent”). Seven items constitute the Parenting Efficacy subscale, which assesses the degree of competence parents feel in their parenting role (e.g., “I honestly believe I have all the skills necessary to be a good parent to my child”). Items are rated on a 6-point scale ranging from *strongly disagree* (1) to *strongly agree* (6), with satisfaction items being reverse-scored. Subscale scores are calculated using the sum score of all items belonging to the respective (sub)scale. Higher scores indicate a higher sense of parenting satisfaction and efficacy. The internal consistency of the PSOC has been established (α = 0.75 and 0.76 for the Satisfaction and Efficacy subscales, respectively) [46].

##### Parenting stress

The Parenting Stress Index—Short Form (PSI-SF [1]) is a 36-item questionnaire comprising three subscales of 12 items each: Parental Distress, Parent–Child Dysfunctional Interaction, and Difficult Child. The Parental Distress subscale assesses the distress a parent experiences due to parenting-related personal factors (e.g., “I often feel tired, worn out, or exhausted from raising a family”). Parent–Child Dysfunctional Interaction measures the dissatisfaction parents feel regarding the interactions with their child (e.g., “I feel that my child isn't meeting my expectations”). The Difficult Child subscale assesses parents’ perception of their child’s manageability (e.g., “My child is more demanding than most children”). Parents rated each item on a 5-point scale ranging from *strongly disagree* (1) to *strongly agree* (5). A sum score is created for each subscale. Higher scores indicate more parenting stress. The internal consistency of the PSI-SF is good (*α*’s ranging from 0.80 to 0.91), test–retest reliability was high (*r*’s between 0.68 and 0.85), and the factor structure supported its validity [1].

##### Parenting style

The Parenting Scale (PS [6]) assesses the degree to which parents use dysfunctional parenting styles towards their child. It comprises three subscales: Laxness (11 items), Overreactivity (10 items), and Verbosity (7 items). The Laxness subscale assesses the degree to which parents give in or allow their child to break rules (e.g., “When my child does something I don't like, I often let it go”). Overreactivity reflects the extent of displayed anger or irritability towards the child (e.g., “I almost always use bad language or curse”). The Verbosity subscale measures to what extent parents talk to their children to solve problems (e.g., “I give my child a long lecture”). Parents rated the occurrence of specific dysfunctional parenting behaviors on a 7-point scale ranging from *never* (1) to *always* (7). A sum score is created for each subscale, with higher scores indicating more use of the concerned dysfunctional parenting style. Internal consistency (*α*’s between 0.63 and 0.84) and test–retest reliability (*r*’s between 0.79 and 0.84) of the PS were adequate [6]. Although the PS was originally developed and validated in samples of parents of preschool-aged children, subsequent research has demonstrated that the measure is psychometrically sound and appropriate for use with parents of older children as well (e.g., [48]).

#### Child-related Measures

##### Psychosocial functioning

The Strengths and Difficulties Questionnaire (SDQ [14]; Dutch translation [45]) was used to assess psychosocial functioning of the child. The SDQ contains 25 items rated on a three-point scale with the following anchors: 0 = not true, 1 = somewhat true, 2 = certainly true (reverse-scored for reverse-formulated items). The items are equally divided over five subscales: emotional symptoms, conduct problems, hyperactivity/inattention, peer relationship problems, and prosocial behavior. The scores for each subscale are the sum of the individual items, resulting in a score from 0 to 10 on each subscale. Higher scores indicate more problems on the first four subscales and more prosocial behavior on the latter subscale. Internal consistency of the Dutch SDQ is acceptable (*α*’s between 0.57 and 0.81 for the parent report version) [45].

##### Adaptive behavior

We assessed the child’s degree of adaptive behavior with the Vineland Adaptive Behavior Scales II (VABS-II [39]). The VABS-II contains 383 items describing behaviors across four subscales: communication (99 items), daily living skills (109 items), socialization (99 items), and motor skills (76 items; only until the age of 6). Given that the study also included children older than 6, the motor skills subscale was not assessed in the current study. Through an interview with parents, the administrator rated the occurrence of the behaviors on a three-point scale (0 = child never or very seldom performs said behavior, 1 = child sometimes performs said behavior, 2 = child usually performs said behavior). All subscales have age-standardized normative scores with a mean of 100 (*SD* = 15). Higher scores indicate more adaptive behavior. Given that no Dutch normative scores are available, we used normative scores of the United States [39]. Internal consistency of the VABS-II was good, based on split-half reliability and the Spearman-Brown prediction formula (across varying ages), *r*’s ranged from 0.84–0.93.84.93 for communication, from 0.86–0.91.86.91 for daily living skills, from 0.84–0.93.84.93 for socialization, and from 0.77–0.90.77.90 for motor skills [39].

### Data Analysis

#### Baseline characteristics

To test for differences between conditions on baseline characteristics, we conducted one-way analyses of variance for the continuous variables, and Chi square tests for the categorical variables, with condition (face-to-face, blended, waitlist) as the independent variable.

#### Baseline to posttreatment

We tested the effects of both parent training formats (versus waitlist) in two separate linear regression analyses for each outcome, leading to a total of 16 regression models. That is, we calculated difference scores (posttreatment – baseline) of each outcome measure to include as the dependent variable. Each parent training format was compared to the waitlist condition separately. Two dummy variables for condition were included in each model: face-to-face (1) versus waitlist (0) and blended (1) versus waitlist (0). In line with Breider et al. (2024), we analyzed data on an intention-to-treat basis: all participants with posttreatment data were included in the analyses, regardless of completion status.

As children’s age may influence both parenting behavior and child (mal)adaptive behavior in children with autism (e.g., [15, 25], also see [8]), we added age as a covariate in all regression models. Moreover, to control for baseline differences and in line with our previous study [8], scores at baseline on the respective outcomes were also included as covariates in each model [43]. We assessed homogeneity of regression slopes by including interaction terms of the independent variables (age × condition dummies and baseline scores × condition dummies) in the model. Significant interactions were kept in the final model after centralizing the respective variable.

#### Baseline to 6-month Follow Up

Given that the waitlist condition started with parent training after the 20-week waitlist period, 6-month follow-up data were only available for the two parent training conditions. Hence, we tested follow up changes within each parent training condition with linear regression analysis, using the difference score (follow up – baseline) of each outcome measure as the dependent variable. We included age and baseline scores as covariates.

All analyses were performed with IBM SPSS Statistics version 27, with *α* = 0.05. We used Benjamini–Hochberg multiple testing correction to control the false discovery rate across the 16 regression models [7]. Adjusted *p*-values are reported.

An a priori power analysis, as described in our primary outcome study [8], showed that 118 participants (40 in the active conditions and 38 in the waitlist) were required to reach 80% power to obtain an effect of 0.65 on the primary outcome measure (based on Aman et al. [3] and Solomon et al. [38]).

## Results

### Baseline characteristics

Baseline descriptives and statistics of the 97 participants are presented in Table 1, separately for each condition. Sex distribution differed between conditions, χ^2^ = 8.44, *p* = 0.015. Post-hoc t-tests revealed that there were significantly more boys in the waitlist condition compared to the face-to-face parent training condition, *p* = 0.004. No other significant differences were found.

**Table 1:** Baseline Descriptives and Statistics

	Face to face (*n* = 33)	Blended (*n* = 33)	Waitlist (*n* = 31)	Statistic	*p*
Child characteristics					
Mean age in years (*SD*)	8.32 (2.39)	8.39 (2.25)	7.88 (2.11)	*F* = 0.46	.634
Male sex *n* (%)	21 (63.6)	26 (78.8)	29 (93.5)	χ^2^ = 8.44	.015
Birth country *n* (%)				χ^2^ = 1.91	.386
The Netherlands	32 (97.0)	32 (97.0)	28 (90.3)		
Other	1 (3.0)	1 (3.0)	3 (9.7)		
School type *n* (%)				χ^2^ = 5.39	.715
Regular primary school	28 (84.4)	25 (75.8)	23 (74.2)		
Special primary school	4 (12.1)	5 (15.2)	3 (9.7)		
Special secondary education		1 (3.0)	3 (9.7)		
Other	1 (3.0)	2 (6.1)	2 (6.5)		
Mean IQ (*SD*)	97.52 (16.86)	99.63 (17.22)	97.67 (15.54)	*F* = 0.16	.853
Mean ADOS-2 comparison score (*SD*)	5.44 (1.88)	5.41 (2.30)	5.48 (1.88)	*F* = 0.01	.988
Comorbid classifications *n* (%)				χ^2^ = 3.47	.483
No comorbid classifications	24 (77.4)	26 (78.8)	23 (74.2)		
ADHD	4 (12.9)	2 (6.1)	6 (19.4)		
Other	3 (9.7)	5 (15.2)	2 (6.5)		
Parent characteristics^1^					
Mean age (*SD*)	38.67 (5.87)	39.35 (6.90)	38.67 (4.86)	*F* = 0.14	.869
Role *n* (%)				χ^2^ = 6.27	.393
Biological mother	30 (90.9)	29 (87.9)	28 (90.3)		
Biological father	3 (9.1)	2 (6.1)	2 (6.5)		
Adoptive mother		2 (6.1)			
Step father			1 (3.2)		
Has job: *n* yes (%)	24 (72.7)	22 (66.7)	18 (58.1)	χ^2^ = 1.54	.463
Hours a week	22.17 (10.59)	24.86 (7.94)	25.83 (12.97)	*F* = 0.69	.508
Has partner: *n* yes (%)	29 (87.9)	26 (78.8)	29 (93.5)	χ^2^ = 3.07	.215

*Note.* IQ = Intelligence Quotient; ADOS = Autism Diagnostic Observation Schedule; ADHD = Attention Deficit Hyperactivity Disorder

^1^Concerns the parent who attended the training and filled out the questionnaires

### Baseline to posttreatment

Compared to the waitlist condition, face-to-face parent training led to a significantly larger increase in parents’ sense of effectiveness as a parent (PSOC Efficacy subscale, *t* = 3.15, *p*_adjusted_ = 0.032) and a larger decrease in using an overreactive parenting style (*t* = −2.94, *p*_adjusted_ = 0.032). No other effects were found for this condition (*t*s < 2.15, *p*s_adjusted_ > 0.187). Parents who received the blended parent training did not show any significant differences on parent-related outcomes compared to parents in the waitlist condition, *t*s < 2.22, *p*s_adjusted_ > 0.240.

We found no significant difference between the groups on any child-related outcome measure, *t*s < 2.44, *p*s_adjusted_ > 0.188. That is, neither the face-to-face nor the blended parent training led to significantly more improvement on psychosocial functioning and adaptive behavior compared to waitlist. For detailed statistics, see Table 2.

**Table 2:** Mean Scores (SD) at Baseline and Posttreatment and Regression Results

									Statistics							
	Mean scores (*SD*)								Baseline vs. posttreatment				Baseline vs. follow-up			
	Face to face			Blended			Waitlist		F2F vs. waitlist		Blended vs. waitlist		Face to face		Blended	
	Baseline	Post treatment	Follow-up	Baseline	Post treatment	Follow-up	Baseline	Post treatment	Test statistic (*t*)	Adjusted *p*-value	Test statistic (*t*)	Adjusted *p*-value	Test statistic (*t*)	Adjusted *p*-value	Test statistic (*t*)	Adjusted *p*-value
Parenting Sense of Competence																
Satisfaction	40.88 (7.14)	43.31 (6.12)	43.71 (5.02)	40.73 (5.29)	42.23 (7.52)	43.52 (7.3)	40.52 (6.08)	39.64 (7.02)	2.15	.187	1.35	.960	5.02	**.008**	0.76	.486
Efficacy	26.91 (5.68)	30.03 (5.29)	31.04 (4.92)	28.73 (4.88)	29.73 (4.00)	31.14 (5.14)	27.45 (3.60)	26.96 (4.95)	3.15	**.032**	2.22	.240	4.02	**.008**	3.74	**.015**
Parenting Stress																
Parental Distress	26.55 (7.06)	24.50 (6.89)	23.04 (6.22)	24.48 (6.66)	25.19 (9.13)	24.67 (9.46)	27.74 (7.56)	26.92 (8.62)	−0.69	.655	0.48	.777	1.86	.109	1.74	.198
Dysfunctional Interaction	25.00 (6.81)	24.16 (7.30)	23.14 (6.43)	26.15 (6.90)	24.15 (7.35)	21.62 (6.89)	26.03 (7.07)	23.76 (6.29)	−0.15	.940	−0.57	.831	1.96	.098	2.81	.027
Difficult Child	36.45 (7.86)	31.38 (7.59)	30.57 (6.83)	37.67 (7.28)	32.42 (9.47)	30.76 (9.3)	36.97 (7.31)	34.44 (6.51)	−1.57	.323	−1.05	.795	3.18	**.013**	1.14	.357
Parenting Style																
Laxness	27.82 (9.83)	24.72 (9.11)	23.64 (8.80)	26.24 (8.36)	25.42 (10.88)	21.89 (6.83)	29.00 (8.28)	30.92 (11.04)	−1.45	.347	−0.62	.999	0.99	.381	1.58	.216
Overreactivity	29.61 (8.56)	23.53 (7.76)	23.21 (7.02)	29.61 (7.67)	28.08 (9.81)	23.11 (4.42)	28.97 (6.79)	30.40 (8.93)	−2.94	**.032**	−0.73	.999	1.33	.242	3.44	**.012**
Verbosity	25.73 (5.78)	22.72 (6.17)	22.54 (5.97)	27.03 (5.49)	25.50 (6.26)	23.32 (4.4)	26.61 (4.80)	26.08 (5.32)	−1.64	.339	−0.34	.843	2.27	.057	1.14	.335
SDQ																
Hyperactivity/inattention	5.91 (3.01)	4.75 (2.84)	4.89 (2.98)	5.38 (2.52)	5.28 (2.64)	4.3 (2.79)	6.83 (2.94)	5.65 (2.91)	−0.39	.801	1.14	.999	1.50	.196	0.97	.392
Emotional symptoms	4.18 (2.44)	3.50 (2.76)	2.78 (2.47)	4.59 (2.86)	3.28 (2.82)	3.26 (2.4)	4.33 (2.96)	3.23 (2.08)	0.02	.982	−1.09	.890	0.32	.750	2.96	**.026**
Conduct problems	3.42 (1.95)	2.41 (1.92)	2.37 (1.90)	3.47 (1.95)	2.76 (2.01)	1.87 (1.79)	3.57 (1.65)	3.31 (2.04)	−1.36	.354	−0.59	.890	2.35	.054	1.25	.326
Peer relationship problems	4.21 (2.18)	2.88 (2.08)	3.11 (2.28)	2.97 (1.99)	2.60 (2.48)	2.35 (1.82)	3.9 (2.29)	3.23 (2.14)	−1.21	.412	0.23	.873	0.37	.761	0.06	.950
Prosocial behavior	5.33 (1.81)	5.94 (2.06)	5.93 (1.92)	5.72 (2.41)	6.04 (2.85)	6.83 (2.41)	5.03 (2.20)	4.96 (1.82)	1.10	.440	0.54	.784	2.82	**.027**	1.68	.194
VABS-II																
Communication	82.81 (10.16)	86.03 (11.88)	83.93 (9.76)	86.03 (11.32)	87.52 (13.27)	89.04 (9.58)	83.50 (12.64)	83.74 (12.26)	1.02	.454	0.62	.956	3.46	**.008**	3.88	**.015**
Daily living skills	86.75 (11.01)	88.00 (11.14)	92.36 (11.97)	89.12 (10.82)	90.03 (12.13)	92.17 (10.66)	88.73 (13.05)	89.96 (14.36)	0.39	.854	0.04	.971	2.38	.057	2.83	**.027**
Socialization	81.13 (12.11)	86.38 (11.14)	86.79 (9.60)	86.06 (12.30)	89.69 (12.29)	88.48 (12.39)	84.30 (11.95)	82.96 (11.79)	2.01	.188	2.44	.272	3.47	**.010**	3.62	**.011**

*Note.* P-values in bold are significant after correction. F2F = Face to face; SDQ = Strengths and Difficulties Questionnaire; VABS = Vineland Adaptive Behavior Scale

In each regression model, we explored the main effects of the included covariates (i.e., child’s age and baseline score of the respective outcome). That is, we looked at the overall effect of these variables across conditions. Child’s age was no significant predictor in any of the models, *t*s < 2.64, *p*s_adjusted_ > 0.160, indicating that the investigated child and parent outcomes were not predicted by the age of the child. Regarding the baseline scores covariate, only baseline emotional problems (SDQ) was a significant predictor of the change from baseline to posttraining. Higher baseline emotional problems led to larger decreases on this subscale, *t* = −3.20, *p*_adjusted_ = 0.032.

### Baseline to 6-month Follow Up

Within-condition analyses from baseline to 6-month follow-up showed that the face-to-face parent training was associated with significant increases in parental satisfaction and parenting self-efficacy, and a decrease in parents’ perception of their child’s manageability (PSI-SF Difficult Child subscale). Blended parent training was associated with an increase in parenting self-efficacy and decreases in dissatisfactory parent–child interactions and the use of an overreactive parenting style. See Table 2 for all baseline to follow-up statistics.

Concerning child-related outcome measures, face-to-face parent training was associated with increases in prosocial behavior (SDQ) as well as communication and socialization skills (VABS-II). Blended parent training was associated with a decrease in the child’s emotional problems (SDQ), and an increase in adaptive behavior (all VABS-II subscales).

## Discussion

In the current study, we investigated parenting and child psychosocial and adaptive functioning outcomes of a randomized controlled trial comparing individual face-to-face and blended behavioral parent training to a waitlist control condition for disruptive behavior in children with autism . We previously found that the face-to-face parent training, but not the blended version, led to a decrease in children’s noncompliance and irritability [8]. Here, we tested whether these improvements extended towards (1) parenting outcomes and (2) child outcomes.

Measured directly post-intervention, face-to-face parent training, but not blended parent training, led to improvements on two of the eight parenting outcomes tested, relative to a waitlist control condition. Specifically, parental self-efficacy increased—indicating that parents felt more confident about managing their parenting tasks and dealing with challenges related to raising their child—and overreactive parenting decreased, suggesting less emotional, intense or harsh responses towards the behavior of their child. Increases in parental self-efficacy are crucial as higher self-efficacy is associated with positive outcomes at many levels: it is linked with parents’ psychological functioning in general and predictive of changes in the behavior of the child [20]. Also, increased parental efficacy has been found to be linked with decreased negative parenting [34], which is in line with the reduction in overreactive parenting we observed. This reduction in overreactive parenting is promising, as literature on parent training for children with behavioral difficulties suggests that this factor is the most robust mediator driving improvements in children (e.g., [9, 11, 19, 31]). For children with autism , there is a lack of such studies, leaving the key mechanisms of changes in parent training for this group unclear.

We found no effects immediately post-intervention on parental satisfaction, parenting stress, or the parenting styles of laxness and verbosity. Interestingly, the lack of impact on parenting stress contrasts with meta-analytical evidence showing a moderate reduction in parenting stress in parents of children with autism and disruptive or hyperactive behavior who received parent training (*d* = 0.37; [41]). However, it should be noted that 6 out of the 7 individual studies within this meta-analysis had a non-significant effect size. This suggests that the effects of parent training on parenting stress might be relatively small, to such an extent that individual studies were underpowered to detect these effects.

For children’s psychosocial functioning, we found no improvement on any of our outcome measures (hyperactivity/inattention, emotional symptoms, conduct problems, peer relationship problems, prosocial behavior, and adaptive behavior). This means that despite the observed improvements in parenting, child behaviors did not change. This was surprising, in particular for conduct problems and hyperactivity/inattention, because these are conceptually related to noncompliance and irritability, on which we did find effects previously [8]. Furthermore, ample research has indicated that decreases in negative parenting practices often predict improvements in child externalizing behavior (e.g., [9, 11, 19, 31]). For the other child outcomes, effects of behavioral parent training might be less logical. To improve peer problems, emotional problems, prosocial behavior and adaptive behavior, other interventions might be indicated, such as naturalistic developmental behavioral interventions [33] or cognitive behavioral interventions [29, 32].

Our within-group analyses on the change from baseline to 6-month follow-up showed more significant results than the between-group findings immediately post-intervention. For the face-to-face condition, three of the eight parenting outcomes tested showed a significant change: parental satisfaction and efficacy improved and parenting stress (specifically how challenging the parents perceived their child) decreased. For the blended condition, two of the eight parenting outcomes tested showed a significant change: parental self-efficacy improved and overreactive parenting decreased. In contrast to the between-group findings immediately post-intervention, within-group analyses from baseline to follow-up also indicated positive changes on some of the child outcomes. For the face-to-face condition, improvements in prosocial behavior, communication, and socialization were observed, and in the blended condition, children showed a decrease in emotional problems and an improvement in all domains of adaptive behavior (i.e., communication, daily living skills, and socialization).

Several limitations of our study warrant consideration. First, our sample consists of relatively high functioning families of children with autism (e.g., average child intelligence levels, the majority of children had no co-occurring mental health problems, most children attended regular education). On the one hand this may limit the generalizability of our findings to families of children with more severe problems, but on the other hand it is reassuring that parent training is also effective in case of low severity of problems.

Second, the main outcome measures presented in this study were parent-reported measures. As parents were also the recipients of the interventions, positive outcomes could indicate an investment or leniency bias (i.e., parents may have reported improvements because they invested a lot, or because they became more tolerant/acceptant towards the behavior of the child). An alternative way to measure outcomes could be by observations of the behavior of the child, but such measures have limited validity and reliability [26]. Most crucially, we opted for parent-reported outcomes measures because of their superior ecological validity.

Third, our study took place prior to the COVID-19 pandemic, at a time when blended intervention formats were less common. Many families in this condition dropped out, indicating low acceptability of the blended intervention. Today, such interventions are likely to be more widely accepted. It is important to note, however, that also before the pandemic many studies already highlighted the potential benefits of digital parent training formats for disruptive behavior (for meta-analyses, see [40, 42]). The lack of effects of our blended program are therefore unlikely to be solely caused by low acceptability of online interventions.

Fourth, our study was powered based on the expected effects on the primary outcome measures, whereas effects on secondary outcomes may be smaller. In addition, we included a slightly lower number of participants than anticipated. Together, this leaves us with the possibility that our study may have been slightly underpowered.

Overall, our findings extend the earlier reported short-term effects of face-to-face behavioral parent training on children’s noncompliance and irritability, by showing that face-to-face parent training improved parenting sense of competence and decreased overreactive parenting. This suggests that the intervention was also beneficial at the level of the parent, which adds to the evidence for face-to-face parent training as an effective intervention for children with autism and disruptive behaviors [30, 41]. Future research should investigate whether improvements in parenting mediate reductions in child disruptive behavior and whether subgroups of children for whom parent training might be particularly helpful or less helpful can be identified. Given the current evidence, however, face-to-face parent training can be encouraged to use in routine youth mental health care for children with autism and disruptive behavior.

## Data Availability

Data can be shared upon reasonable request.

## Footnotes

1 Note that we use person-first instead of identity-first language throughout this manuscript, as this is preferred by most people with autism in Dutch (de Laet et al., 2023).
